# Model selection for dynamical systems via sparse regression and information criteria

**DOI:** 10.1098/rspa.2017.0009

**Published:** 2017-08-30

**Authors:** N. M. Mangan, J. N. Kutz, S. L. Brunton, J. L. Proctor

**Affiliations:** 1 Department of Applied Mathematics, University of Washington, Seattle, WA 98195, USA; 2 Institute for Disease Modeling, Bellevue, WA 98005, USA; 3 Department of Mechanical Engineering, University of Washington, Seattle, WA 98195, USA

**Keywords:** model selection, information criteria, sparse regression, nonlinear dynamics, data-driven discovery

## Abstract

We develop an algorithm for model selection which allows for the consideration of a combinatorially large number of candidate models governing a dynamical system. The innovation circumvents a disadvantage of standard model selection which typically limits the number of candidate models considered due to the intractability of computing information criteria. Using a recently developed sparse identification of nonlinear dynamics algorithm, the sub-selection of candidate models near the Pareto frontier allows feasible computation of Akaike information criteria (AIC) or Bayes information criteria scores for the remaining candidate models. The information criteria hierarchically ranks the most informative models, enabling the automatic and principled selection of the model with the strongest support in relation to the time-series data. Specifically, we show that AIC scores place each candidate model in the *strong support*, *weak support* or *no support* category. The method correctly recovers several canonical dynamical systems, including a susceptible-exposed-infectious-recovered disease model, Burgers’ equation and the Lorenz equations, identifying the correct dynamical system as the only candidate model with strong support.

## 1. Introduction

**Figure 1.**
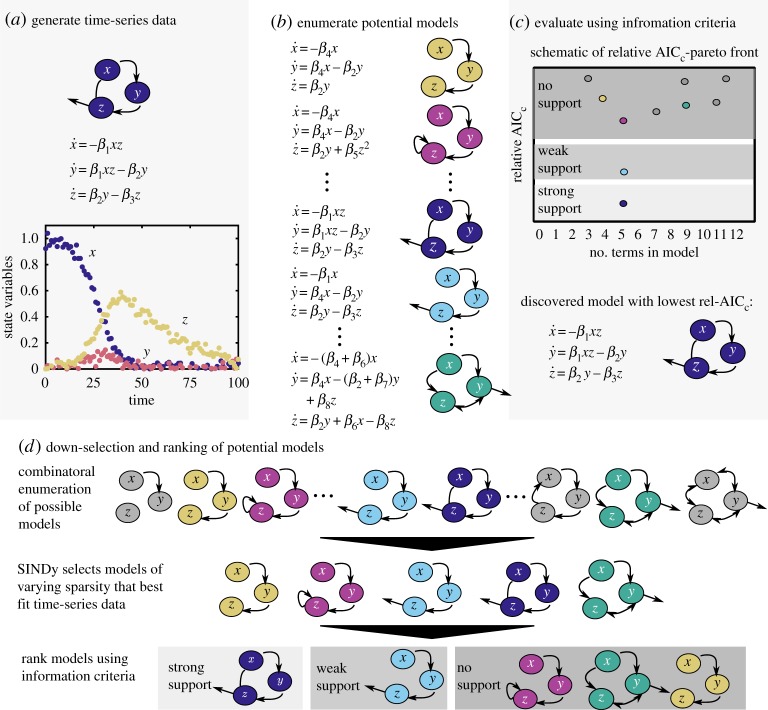
Schematic of model selection process, with (*a*) data generation, (*b*) generation of a set of potential models and (*c*) comparison of the models as a function of the number of terms in the model and relative Akaike information criteria (AIC_c_). Section (*d*) shows how models are down-selected from a combinatorially large model space using SINDy and then further sub-selected and ranked using information criteria. (Online version in colour.)

Nonlinear dynamical systems theory has provided a fundamental characterization and understanding of phenomenon across the physical, engineering and biological sciences. Traditionally, simplified models are posited by domain experts, and simulations and analysis are used to explore the underlying dynamical behaviour which may include chaotic dynamics (e.g. Lorenz equations), nonlinear oscillations (e.g. van der Pol, Duffing) and/or bifurcations. The emergence of data-driven modelling methods provides an alternative framework for the discovery and/or inference of governing nonlinear dynamical equations. From this perspective, governing models are posited from time-series measurement data alone. The recent *sparse identification of nonlinear dynamics* (SINDy) method [1] uses sparse regression and a Pareto analysis to correctly discover parsimonious governing equations from a combinatorially large set of potential dynamical models. This methodology can be generalized to spatio-temporal systems [2,3] and dynamical systems characterized with rational function nonlinearities which often occur in biological networks [4]. Although previously suggested [4], no explicit connection between the SINDy process and information theoretic criteria has been established. Information criteria are the standard statistical method established for the model selection process. In this manuscript, we demonstrate that the Akaike information criteria (AIC) can be connected with the SINDy architecture to hierarchically rank models on the Pareto front for automatic selection of the most informative model. As outlined in figure 1, the AIC scores can be used to correctly infer dynamical systems for a given time-series dataset from a combinatorially large set of models. To our knowledge, this is the first explicit demonstration of how information theory can be exploited for the identification of dynamical systems.

**Figure 2.**
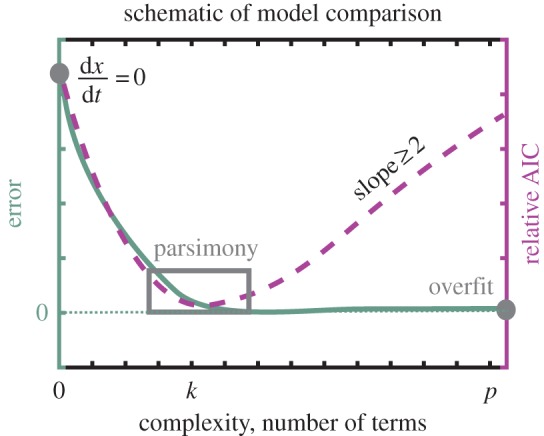
Schematic of Pareto front for evaluatingthe number of terms (*x*-axis) versus the error (left axis, solid green), and the number of terms versus the AIC score (right axis, dashed magenta). Left grey dot indicates a high-error model with zero terms (*dx*/*dt*=0). Grey box shows the region of parsimonious models balancing error and complexity. Right grey dot indicates an overfit model, which can produce zero error. Note that the standard AIC score has an asymptotic penalty of 2*k* for the number of terms, resulting in a slope of at least 2 for large *k*. (Online version in colour.)

Successful model identification inherently requires a rigorous method for validation and comparison. Model selection procedures found in the literature (i.e. [1,5,6]) typically rely on a Pareto analysis, which balances accuracy and model complexity. Figure 2 illustrates this trade-off. As the solid-green line (left axis) indicates, the error for a dynamical system model with zero terms (*dx*/*dt*=0) is high. Increasing the complexity of the model, by adding terms, provides a better fit to the data. As the number of terms in the model approaches the number of free parameters, one can guarantee the error will approach zero. However, overfitting to data, especially in the presence of noise, produces models that poorly predict the behaviour of validation experiments (out-of-sample data). The overtraining and over-completeness of models are critical concerns in machine-learning-methods. One generally seeks to identify parsimonious models (grey box in figure 2) where the error is significantly reduced using the minimal number of terms. Parsimony not only avoids overfitting to training data, but also reflects an Occam’s razor approach, which is generally preferred in physical and biological modelling. Unfortunately, interpreting the Pareto analysis is often ambiguous. The Pareto front may not have a sharp elbow but may instead have a cluster of models near the elbow.

Information theory provides a rigorous statistical framework for selecting a model from a set of candidate models given validation data. As early as the 1950s, a measure of information loss between empirically collected data and model-generated data was proposed to be computed using the Kullback–Leibler (KL) divergence [7,8]. Akaike built upon this notion to establish a *relative* estimate of information loss across models that balances model complexity, and goodness-of-fit [9,10]. This allowed for a principled model selection criteria through the AIC. The AIC was later modified by G. Schwarz to define the more commonly used Bayes information criteria (BIC) [11]. Both AIC and BIC compute the maximum log likelihood of the model and impose a penalty: AIC adds the number of free parameters *k* of the posited model, while BIC adds half of *k* multiplied by the log of the number of data points *m*. This penalty increases the information criteria score for larger, overfit models, creating a minimum in the AIC curve and allowing for more intuitive model selection, as illustrated in figure 2. Much of the popularity of BIC stems from the fact that it can be rigorously proved to be a consistent score [11]. Thus, if a number of models *q* are proposed, with one of them being the true model, then, as m→∞, the true model has the lowest score with probability approaching unity. Regardless of the selection criterion, AIC or BIC, they both provide a relative estimate of information loss across a selection of *q* models, quantitatively balancing model complexity and goodness-of-fit [12].

Although successful and statistically rigorous, model selection in its standard implementation is typically performed on *q* predetermined candidate models, where *q* is often 10 or less [12–17]. For modern applications to dynamical systems where rich, high-fidelity time-series data can be acquired, the restriction on the number of models limits the potential impact of AIC/BIC scores for discovering the correct nonlinear dynamics. Instead, it is desired to consider a combinatorially large set of potential dynamical models as candidates, thus enforcing that *q*≫1. This is computationally intractable with standard model selection, as each of the models from the combinatorially large set would have to be simulated and then evaluated for a AIC/BIC score.

As an alternative, sparse regression techniques, embodied by the *lasso* (least absolute shrinkage and selection operator) method of Tibshirani [18], have enabled variable selection algorithms capable of optimally choosing among a combinatorially large set of potential predictors. Specifically, a lasso regression analysis, or one of its many generalizations and variants, performs both variable selection and regularization in order to enhance the prediction accuracy and interpretability of the statistical model it produces. Such mathematical tools provide a critically enabling framework for model selection, in particular, for identifying dynamical systems.

In this work, we demonstrate a new mathematical framework that leverages information criteria for model selection with sparse regression for evaluating a combinatorially large set of candidate models. Specifically, we circumvent a direct computation of information criteria for the combinatorially large set of models by first sub-selecting the candidate functional forms that are most consistent with the time-series data. Thus, we integrate two maturing fields of statistical analysis: (i) sparse regression for nonlinear systems identification via SINDy and (ii) model selection via information criteria. Our algorithm is demonstrated to produce a robust procedure for discovering parsimonious, nonlinear dynamical systems from time-series measurement data alone. We demonstrate the methodology on a number of important examples, including the susceptible-exposed-infectious-recovered (SEIR) disease model, the Burgers’ partial differential equation (PDE) and the Lorenz equations, and demonstrate its efficacy as a function of noise, length of time series and other key regression factors. Our sparse selection of dynamical models from information theory criteria ranks the candidate models and further shows that the correct model is strongly supported by the AIC/BIC scores. Ultimately, the method provides a cross-validated and ranked set of candidate nonlinear dynamical models for a given time series of measurement data, thus enabling data-driven discovery of the underlying governing equations.

## 2. Background

### (a) Model selection via information criteria

The process of model selection fundamentally enables the connection of observations or *data* to a mathematical model. Further, a well-selected model, which describes a governing law or physical principle underlying the system process, can be used for prediction outside of the sampled data and parameter configuration [12]. The substantial challenge facing the selection process is discovering the *best* predictive model from a combinatorially large space of available models. To emphasize the enormity of this task, consider the number of possible polynomial models up to degree 4 with five state variables. Approximately 10^38^ models would need to be constructed, fit to the data and compared according to goodness-of-fit [4]. Thus, model selection quickly becomes computationally intractable for a modest number of variables and polynomial degree.

Typically, a sub-selection of models occurs based on prior scientific knowledge of the process to produce a subset, O(10), of heuristically defined *candidate models* [12–17]. Recent research has focused on automatically expanding the number of candidate models [6,19,20]. Once a subset of models is chosen, the model selection procedure balances the goodness-of-fit with model complexity, i.e. the number of free parameters. A wide variety of rigorous statistical criteria have been developed to balance model parsimony and predictive power including popular methods such as the Akaike information criterion (AIC) [9,10], Bayesian information criterion (BIC) [11], cross validation (CV) [21], deviance information criterion (DIC) [22] and minimum description length [23]. Methods such as AIC explicitly balance parsimony and relative information loss across models, penalizing the number of parameters in the model to avoid overfitting.

In this manuscript, we use the ubiquitous and well-known AIC as the statistical criterion for comparing candidate models. The AIC value for each candidate model *j* is

2.1 $${\mathrm{AIC}}_{j}=2k-2\ln(L(\text{x},\hat{\mu })),$$

where *L*(**x**,*μ*)=*P*(**x**|*μ*) is the likelihood function (conditional probability) of the observations **x** given the parameters *μ* of a candidate model, *k* is the number of free parameters to be estimated and $\hat{\mu }$ is the best-fit parameter values for the data [9,10]. Note that the penalty, 2*k*, enforces a lower bound on the relative AIC scores; figure 2 illustrates the general shape of AIC scores as the number of free parameters (terms) increases. In practice, the AIC requires a correction for finite sample sizes given by

2.2 $${\mathrm{AIC}}_{c}=\mathrm{AIC}+\frac{2(k+1)(k+2)}{\left(m-k-2\right)},$$

where *m* is the number of observations. A common likelihood function uses the residual sum of squares (RSS), given by $\mathrm{RSS}=\sum_{i=1}^{m}{\left(y_{i}-g(x_{i};\mu )\right)}^{2}$, where *y*_*i*_ are the observed outcomes, *x*_*i*_ are the observed independent variables and *g* is the candidate model. The RSS is a well-known objective function for least-squares fitting. In this case, AIC can be expressed as AIC=mln⁡(RSS/m)+2k [12]. Note that (2.1) penalizes, by increasing the AIC score, the models that have a large number of free parameters and which are unable to capture the characteristics of the observed data.

### (b) Sparse identification of nonlinear dynamics and sparse model selection

Identifying dynamical systems models from data is increasingly possible with access to high-fidelity data from simulations and experiments. With traditional methods, only a small handful of model structures may be posited and fit to data via regression. Indeed, simultaneous identification of both the structure and the parameters of a model generally requires an intractable search through combinatorially many candidate models. Genetic programming has been recently used to determine the structure and parameters of dynamical systems [6,24,25] and control laws [26], enabling the efficient search of complex function spaces. Sparsity-promoting techniques have also been employed to simultaneously identify the structure and parameters of a dynamical system model. More broadly, there is a considerable body of work in the control literature on nonlinear system identification with explicit connections to AIC and BIC [27–29]. Nonlinear autoregressive moving average models with exogenous inputs (NARMAX models) have also been used for systems identification in conjunction with information criteria [30,31]. Compressed sensing was first used to determine the active terms in the dynamics [32], although it does not work well with overdetermined systems that arise when measurements are abundant. By contrast, the SINDy algorithm [1] uses sparsity-promoting regression, such as the *lasso* [18] or sequential thresholded least-squares algorithm [1], to identify nonlinear dynamical systems from data in overdetermined situations.

Here, we review the SINDy architecture for identifying nonlinear dynamics from data. The general observation underlying SINDy is that most dynamical systems of a state $\text{x}\in R^{n}$,

2.3 $$\frac{d}{dt}\text{x}(t)=\text{f}(\text{x}(t)),$$

have only a few active terms in the dynamics, making them *sparse* in a suitable function space. To identify the structure and parameters of the model, a set of candidate symbolic functions are first concatenated into a library ***Θ***(**x**)=[*θ*_1_(**x**) ··· *θ*_*p*_(**x**)]. With time-series data $\text{X}\in R^{m\times n}$, where each row is a measurement of the state **x**^T^(*t*_*k*_) in time, it is possible to evaluate the candidate function library $\Theta (\text{X})\in R^{m\times p}$ at the *m* time points. Finally, with derivative data $\dot{\text{X}}\in R^{m\times n}$, either measured or obtained by numeric differentiation, it is possible to pose an optimization problem satisfying the dynamic relationship:

2.4 $$\dot{\text{X}}=\Theta (\text{X})\Xi .$$

The few active terms in the dynamics, given by the non-zero entries in the columns of ***Ξ***, may be identified using sparse regression. In particular, the sparsest matrix of coefficients ***Ξ*** is determined that also provides a good model fit, so that $∥\dot{\text{X}}-\Theta (\text{X})\Xi {∥}_{2}$ is small. Sparse regression has the added benefit of avoiding overfitting, promoting stability and robustness to noise. Since the original SINDy method, there have been numerous innovations and extensions to handle rational function nonlinearities [4], PDEs [2] and highly corrupted data [33], and to build Galerkin regression models in fluids [34]. The method is also connected to the dynamic mode decomposition [35] if only linear functions are used in ***Θ***.

In most sparse regression algorithms, there is a parameter that determines how aggressively sparsity is promoted. The successful identification of the model in equation (2.3) hinges on finding a suitable value of this sparsity-promoting parameter. Generally, the parameter value is swept through, and a Pareto front is used to select the most parsimonious model. However, the Pareto frontier may not have a sharp elbow or may instead have a cluster of models near the elbow, thus compromising the automatic nature of the model selection using SINDy alone.

## 3. Material and methods

Our algorithm integrates sparse regression for nonlinear system identification with model selection via information criteria. This approach enables the automatic identification of a single best-fit model from a combinatorially large model space. In the first step of the algorithm, the SINDy method provides an initial sub-selection of models from a combinatorially large number of candidates. The sub-selection of candidate models *near* the Pareto frontier is critically enabling as it is computationally intractable to simulate and compare against the time-series data for all possible models. Importantly, the sub-selection can take the number of candidate modes from 10^9^ (for our two-dimensional cubic example) to a manageable 10^2^. This then allows for a tractable computation of AIC or BIC scores for the remaining candidate modes. The information criterion hierarchically ranks the most informative models, enabling the automatic selection of the model with the strongest support. This is in contrast with a standard Pareto front analysis which looks for a parsimonious model at the elbow of the error versus complexity curve. Algorithm , using the AIC as our information criteria, is executed for model selection. Figure 1 illustrates the algorithm.

When evaluating dynamical systems models, there is some ambiguity about what constitutes an ‘observation’. We take time-series data for a given set of initial conditions to be an observation, rather than taking each measurement at each time point. To obtain a representative error for the *i*th time-series observation, we calculate the average absolute error over the entire time series: $E_{\text{avg}}=\sum_{\tau }|y_{i}^{\tau }-g(x_{i}^{\tau };\mu )|$. We then substitute this representative error in for (*y*_*i*_−*g*(*x*_*i*_;*μ*)) in the RSS calculation, and take the sum of the squares so that $\mathrm{AIC}=m\ln((\sum_{i=1}^{m}E_{\mathrm{avg}}(x_{i},y_{i}))/m)+2k$. We use this value for AIC in (2.2).

Algorithm 1 requires two sets of time-series data: a relatively small training set $\text{X}\in R^{m\times n}$, used to build the library ***Θ***, and a larger validation set $\text{Y}\in R^{l\times n}$. For each numerical example in §4, both the training and validation sets contain additive Gaussian noise applied at each time-series point, with mean zero and standard deviation *ϵ* = 10^−4^ unless otherwise noted. For the models used in our examples, we can exactly calculate $\dot{\text{X}}$. In practice, the time derivative would need to be calculated numerically from the noisy time-series data. SINDy has successfully been used in combination with total variation regularized differentiation [1], a method for computing derivatives in the presence of noise.


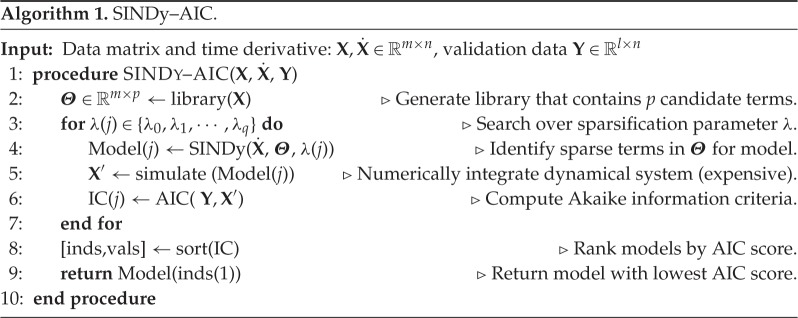


**Figure 3.**
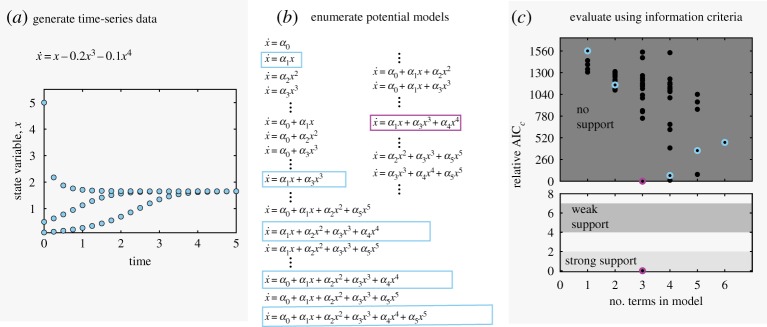
Selectionof model for single variable, *x*, polynomial system. (*a*) Three computationally generated time series with additive noise *ϵ*=0.001. (*b*) Combinatorial model possibilities with those selected by SINDy in boxes. (*c*) Relative AIC_c_ criteria for all possible models (black dots), and those found by SINDy (blue circles). Magnification in lower plot shows strongly and weakly supported AIC_c_ ranges, containing only the correct model (lowest/magenta circle). (Online version in colour.)

**Figure 4.**
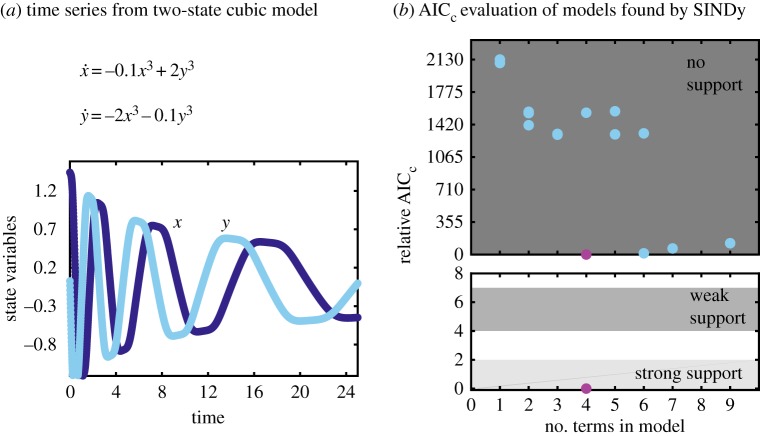
Evaluation of SINDy selected modelsfor two-dimensional cubic system. (*a*) Computationally generated time series from single set of initial conditions with additive noise *ϵ*=0.001. (*b*) Relative AIC_c_ criteria for models found by SINDy (blue circles). Magnification in lower plot shows that strongly and weakly supported AIC_c_range contain only the correct model (lowest/magenta circle). (Online version in colour.)

**Figure 5.**
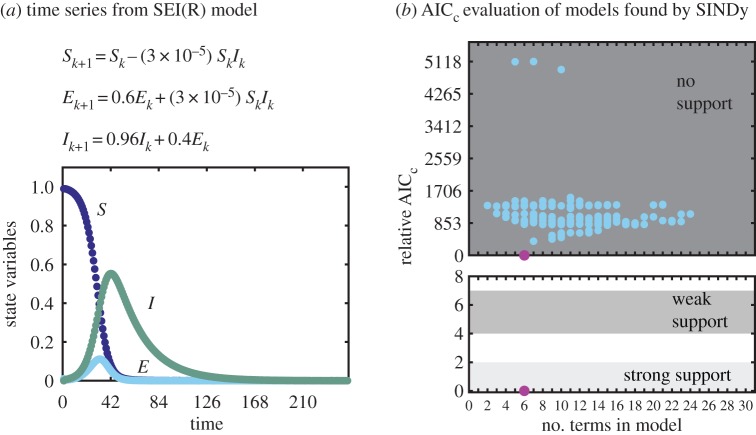
Evaluation of SINDy-selected models for the three state variable SEIR model. (*a*) Computationally generated time series with additive noise*ϵ*=2.5×10^−4^. (*b*) Relative AIC_c_ criteria for models found by SINDy. Magnification in the lower plot shows that strongly and weakly supported AIC_c_ ranges contain only the correct model (lowest/magenta circle). (Online version in colour.)

**Figure 6.**
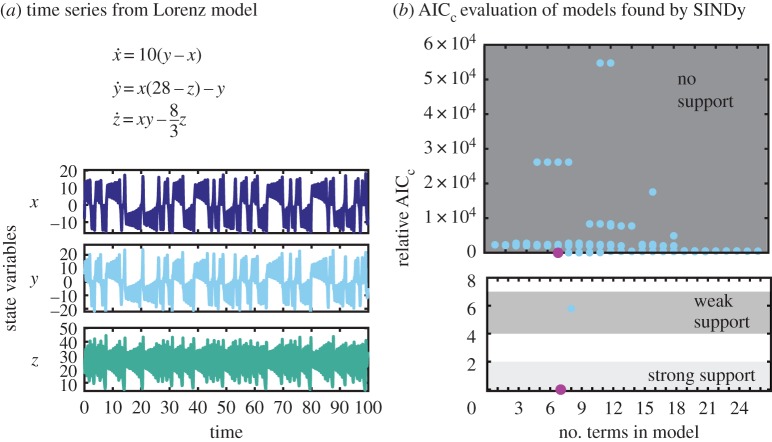
Evaluation of SINDy-selected models for the three state variable Lorenz model. (*a*) Computationally generated time series with additive noise *ϵ*=0.001. (*b*) Relative AIC_c_ criteria for models found bySINDy. Magnification in the lower plot shows strongly and weakly supported AIC_c_ ranges. The correct model (lowest/magenta circle) lies in the strong support range and amodel with an additional small term in the weak support range. (Online version in colour.)

The training sets used for each example are shown in figures 3–6. The time-series instances in each validation set are the same length and have the same sampling frequency as the respective training set, but are initialized at 100 new values. Thus, *l*=100×(*sampling* *rate*)×(*duration* *per* *instance*). The training set **X** is used in step 2 of Algorithm to build the library for sparse inference. The validation set **Y** is used in step 6 of Algorithm to compute the AIC score.

The candidate models with the lowest scores are ranked as the most probable. To be more precise, the AIC scores for each candidate model can have a wide range of values which require a rescaling by the minimum AIC value (AIC_min_) [12,36]. The rescaled AIC values Δ_*j*_=AIC_*i*_−AIC_min_ can be directly interpreted as a strength-of-evidence comparison across models. Models with Δ_*j*_≤2 have *strong support*, 4≤Δ_*j*_≤7 have *weak support* and Δ_*j*_≥10 have *no support* [36]. In principle, the relative-AIC_c_ score can be related to the *p*-value for the statistical support of each model for a particular dataset and number of validation time series, but Burnham & Anderson[12,36] suggest the value we use here as a rule of thumb. These rankings allow for a principled procedure for retaining or rejecting models within the candidate pool of models. For time-series data with enough data samples, high signal to noise and/or sufficiently large set of candidate models, only one model is typically strongly supported by AIC scores. In the examples used to demonstrate the method, the only strongly supported candidate model is, in fact, the correct model.

## 4. Results: model selection

### (a) One-dimensional polynomial model

We demonstrate the relationship between SINDy model selection and AIC ranking procedure, as illustrated in figure 1*d*, with a simple single state variable model with three polynomial terms,

4.1 $$\dot{x\;}\;\;=x-0.2x^{3}-0.1x^{4}.$$

We compute three time series for this model, as shown in figure 3*a*.

Assuming a feature library with up to fifth order polynomials, or *d*=6, there are ∑i=16(6i)=63 possible models. A representation of the combinatorial set of models is shown in figure 3*b*, and we perform least-squares fitting for the coefficients of each model. All models are cross-validated using 100 initial conditions, and relative AIC_c_ scores are calculated. This combinatorially complete set of models (black dots) populate the AIC_c_ Pareto front in figure 3*c*. The relative AIC_c_ score successfully characterizes the true, 3-term model as the only model with ‘strong support.’ All other models within the full combinatorial space fall within the ‘no support’ range.

SINDy enables us to sub-select a set of models from the feature library and, for this simple system, we can compare against the combinatorial set. The models selected by SINDy are boxed in figure 3*c*,*d*. SINDy finds the correct 3-term model, as well as models with 1, 2, 4, 5 and 6 terms. These models and coefficients exactly match a subset of those found by combinatorial least squares, which is expected given that this implementation of SINDy uses least-squares to fit the coefficients. Notably, the 1- and 2-term models selected by SINDy are the most meaningful reductions of the true underlying system (with smaller coefficients set to zero), rather than the 1- and 2-term models with the lowest error in the full feature space.

### (b) Two-dimensional cubic model

For larger systems, enumerating all possible models represented in a given feature library would be computationally infeasible, but by using the SINDy procedure the most relevant models are generated and selected. For example, consider the relatively simple example of a two state variable system (*n*=2) with a fifth order polynomial library (*d*=6). In this case, there are $Nm=\left(\begin{array}{c} n+d\\ d \end{array}\right)=28$ possible monomials, and Np=∑i=1Nm(Nmi)=268,435,455 potential models. Figure 4 demonstrates the results of performing SINDy and AIC evaluation on a cubic model.

With only a single time series for each state variable (*x* and *y*) as input (Figure 4*a*), the SINDy-selected models with varying number of terms (blue circles) include the correct model (lowest/magenta circle). Using 100 randomly selected initial conditions for validation, relative AIC_c_ ranks the correct model as strongly supported, and all other models as having no support.

### (c) Three-dimensional disease transmission model

Next, we apply our method to the SEIR disease transmission model. Models of this type are often used to determine disease transmission rates, detect outbreaks and develop intervention strategies. However, generating the appropriate model for interactions between different populations is currently done heuristically and then evaluated using information criteria [17]. Using SINDy with AIC evaluation would provide *data-driven* model generation and selection from a wider library of possible interaction terms. As a first step, we apply SINDy with AIC to a discrete, deterministic SEIR model as shown in figure 5.

We input a single time series representing an outbreak for the *S*, *E* and *I* state variables (*n*=3), and provide a library of polynomial terms up to second order (*d*=3). For this example, the total number of models represented in the library is Np=∑i=1Nm(Nmi)=1023 with $Nm=\left(\begin{array}{c} n+d\\ d \end{array}\right)=10$ possible monomials. A complication of the SEIR system is that *R* is a redundant state variable; *S*, *E* and *I* have no dependence on *R*, and *R* depends only on a term already represented in the *I*_*k*+1_ equation *R*_*k*+1_=*R*_*k*_+0.04*I*_*k*_. A result of this redundancy, SINDy cannot find the correct equations with *R* included in the library. Without *R*, SINDy selects a set of models from 1023 possible in the library (blue circles), and with 100 validation measurements the relative-AIC_c_ evaluation ranks only the correct 6-term model as having any support (magenta circle) in figure 5*b*.

### (d) Partial differential equation: Burgers’ equation

To demonstrate the applicability of the model selection and ranking with the AIC framework, we apply our methodology to Burgers’ equation. In this case, PDE-FIND, demonstrated by Rudy *et al.* in [2], sparsely selects a set of seven PDEs as given in table 1. We use the same simulated, spatially resolved time series training set and code as in [2]. We alter the sparsity search by decreasing the tolerance to *d*_*tol*_=0.1 and increasing the number of iterations to 50 in the TrainSTRidge algorithm. We evaluate all models found by this more finely resolved sparsity search. Given the increased numerical difficulty of simulating PDEs compared with ODEs, sparse selection of potential models to reduce validation simulations is even more essential.

**Table 1.** PDE-FIND discovered models for Burger’s equation with relative AIC_c_ score.

PDE found	no. terms	ΔAIC_c_
$\begin{array}{ll} u_{t} & =+(0.027)u-(0.109)u^{2}+(0.131)u^{3}\\ & \;-(0.010)u_{x}-(1.010)uu_{x}+(0.535)u^{2}u_{x}\\ & \;-(0.656)u^{3}u_{x}+(0.067)u_{xx}+(0.248)uu_{xx}\\ & \;-(0.485)u^{2}u_{xx}+(0.343)u^{3}u_{xx}\\ & \;-(0.005)u_{xxx}+(0.029)uu_{xxx}\\ & \;-(0.020)u^{2}u_{xxx}-(0.027)u^{3}u_{xxx} \end{array}$	15	333
$\begin{array}{ll} u_{t} & =+(0.025)u-(0.103)u^{2}+(0.125)u^{3}\\ & \;-(0.012)u_{x}-(1.059)uu_{x}+(0.391)u^{2}u_{x}\\ & \;-(0.521)u^{3}u_{x}+(0.068)u_{xx}+(0.238)uu_{xx}\\ & \;-(0.451)u^{2}u_{xx}+(0.311)u^{3}u_{xx}-(0.005)u_{xxx}\\ & \;+(0.035)uu_{xxx}-(0.047)u^{2}u_{xxx} \end{array}$	14	342
$\begin{array}{ll} u_{t} & =+(0.022)u-(0.084)u^{2}+(0.100)u^{3}\\ & \;-(1.160)uu_{x}+(0.630)u^{2}u_{x}-(0.677)u^{3}u_{x}\\ & \;+(0.070)u_{xx}+(0.237)uu_{xx}-(0.462)u^{2}u_{xx}\\ & \;+(0.317)u^{3}u_{xx}-(0.005)u_{xxx}+(0.037)uu_{xxx}\\ & \;-(0.048)u^{2}u_{xxx} \end{array}$	13	355
$\begin{array}{ll} u_{t} & =-(1.074)uu_{x}+(0.386)u^{2}u_{x}-(0.446)u^{3}u_{x}\\ & \;+(0.085)u_{xx}+(0.125)uu_{xx}-(0.262)u^{2}u_{xx}\\ & \;+(0.186)u^{3}u_{xx}+(0.026)uu_{xxx}-(0.043)u^{2}u_{xxx} \end{array}$	9	360
$\begin{array}{ll} u_{t} & =-(1.022)uu_{x}+(0.087)u_{xx}+(0.096)uu_{xx}\\ & \;-(0.097)u^{2}u_{xx} \end{array}$	4	351
*u*_*t*_=−(1.064)*uu*_*x*_+(0.571)*uu*_*xx*_−(0.593)*u*^2^*u*_*xx*_	3	683
*u*_*t*_=−(1.010)*uu*_*x*_+(0.103)*u*_*xx*_	2	0
ground truth: *u*_*t*_=−*uu*_*x*_+(0.1)*u*_*xx*_	2	—

For validation, we run simulations with the same spatial and temporal sampling as the training data, starting at 100 new initial conditions defined by varying amplitude and location of the initial gaussian distribution, *u*_0_. The relative AIC_c_ is calculated by taking the average absolute error (see Material and methods) over all points in time and space for each instance, and using the number of terms or free parameters as indicated in table 1. Only the correct model receives a relative AIC_c_<2 in the strong support regime. The rest have no support.

### (e) Lorenz model

As a final example, we demonstrate SINDy with AIC on the chaotic Lorenz system [37]. Using a library of polynomials up to second order (*d*=3) for the three state variable system (*n*=3), there are once again *N*_*p*_=1023 models represented in the function library. Providing one time series for each state variable, as shown in figure 6*a*, SINDy recovers a subset of these models (circles in figure 6*b*). Using 100 validation measurements on each model, the relative AIC_c_ criteria ranks the correct 7-term model as having strong support (magenta circle). Unlike in previous examples, one other model is ranked as having ‘weak support’ (blue circle). This model has an additional small constant term in the equation for *x*: $\dot{x\;}\;\;=8.5\times 10^{-6}+10(y-x)$.

A possible explanation for the level of support for this model is the chaotic nature of the Lorenz system. Even when the recovered model is correct, small variations in the recovered coefficients (≈10^−6^ for this case) will cause the calculated time series for the recovered model to diverge from the ‘true’ model after some length of time (greater than 1 unit time for these parameters). For the example in figure 6*b*, the validation uses time series of length *t*=5 (arb. units). In a true model-selection situation, we would not know this characteristic length scale ahead of time, and a sensitivity analysis would need to be performed. We discuss this and other challenges to practical implementation in the next section.

## 5. Practical implementation: noise and number of measurements

**Figure 7.**
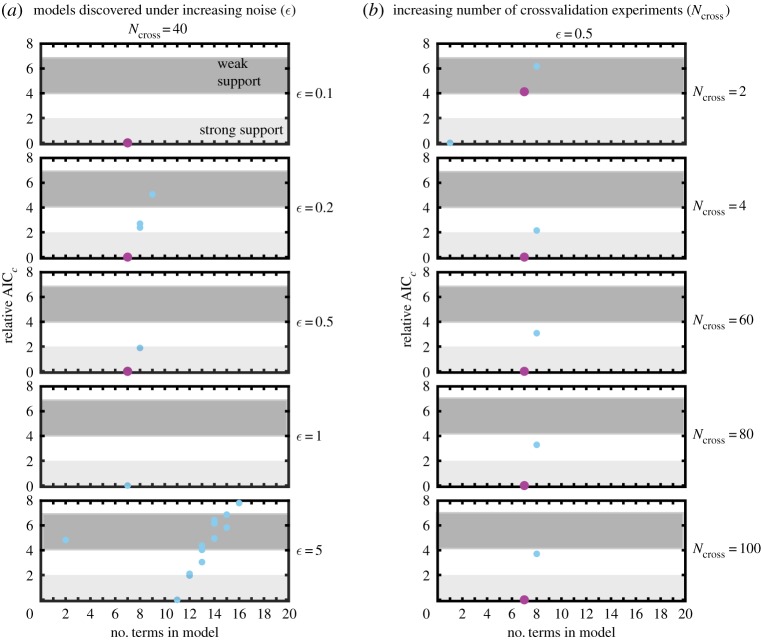
Evaluation of SINDy selected models for three state variable Lorenz model under varying (*a*) noise conditions and using (*b*) increasing number of validation experiments to calculate AIC_c_. Strong and weakly supported relative AIC_c_ range is shown. (Online version in colour.)

SINDy with AIC ranking can successfully select the correct model for a variety of known systems, given low enough measurement noise and a large amount of data for validation. Under practical conditions, the signal-to-noise ratio may be lower than desired and the amount of data available for validation may be restricted. In figure 7, we show the effects of increasing noise and number of validation experiments on the selection of the correct model for the Lorenz system. Reading from the top of figure 7*a*, for low noise, *ϵ*=0.1, only the correct model (magenta) falls within the supported (strong or weak) range. Increasing the noise to *ϵ*=0.2 and 0.5 causes other models to descend into the weak support regime and eventually into the strong support range. Around this level of noise, the relative AIC_c_ scores for the incorrect models are very sensitive to the random additive noise. Repeating the computation for different instances of randomly generated measurement noise results in fluctuation of the AIC_c_ scores for these models (data not shown), although the true model maintains the lowest score (over 10 instances). This suggests that data sub-sampling could be used to test for models with noise-fitted terms.

Above a certain level of noise (*ϵ*=1), the method is unable to robustly select the correct model, and for even higher levels of noise (*ϵ*=5) a larger number of incorrect models appear to have support. For the particular case of the Lorenz system used here, SINDy is unable to select the correct model, and therefore the true model is not evaluated by AIC_c_ at these noise levels. As we are using a relative AIC_c_ score, the score of the lowest model will always be zero, even when the actual error between that model and the data is very high. These examples highlight the importance of examining the error between the model-generated time series against and the data in addition to the relative AIC_c_ score.

The number of time series used for validation also has an impact on the relative AIC_c_ score between the true model and other models given strong support as shown in figure 7*b*. For *ϵ*=0.5 and only two time series used for validation and AIC_c_ calculation, the correct model is selected by SINDy, but a simpler (and incorrect) 1-term model is assigned a lower score. Given only two more time series for validation, (*N*_cross_=4), the correct model has the lowest score. Increasing the number of validation measurements used for evaluation to *N*_cross_=60,80 and 100 increases the relative score of the incorrect 8-term model to the true 7-term model.

The success of the method also relies on the duration and sampling of the time series, especially in the case of chaotic systems like the Lorenz model. Here, the validation experiments run from *t*=0 to *t*_end_=1 (arb. units), as opposed to those in figure 6, which ran to *t*_end_=5. Even with higher noise (*ϵ*=0.1 compared to *ϵ*=0.001), only the true model is supported. Again, sensitivity to sub-sampling of data can help differentiate between noise-fitting and mechanistically essential terms.

## 6. Discussion and conclusion

The integration of mathematical techniques advocated here, (i) sparse regression for nonlinear systems identification via SINDy and (ii) model selection via information criteria, provides a new paradigm for model selection of dynamical systems. Algorithmically, the critical methods combine as follows. In the first step of the algorithm, the SINDy method, which is based upon sparse regression, provides an initial sub-selection of models from a combinatorially large number of candidate models. The selection of candidate models is critically enabling as it reduces the number of potential models to a manageable number, which can each be evaluated through simulation and comparison to the time-series data. Indeed, the remaining candidate models, which are now on the order of 10 models, are each evaluated using information criteria such as AIC or BIC.

The candidate models with the lowest scores are ranked as the most likely. Specifically, in what follows, we work with the AIC score and show that these scores place each candidate model in the *strong support*, *weak support* or *no support* category. For time-series data with enough data samples, a large enough signal to noise and/or a sufficiently large set of candidate models, only one model is typically strongly supported by the AIC score. In the examples used to demonstrate the method, the only strongly supported candidate model is, in fact, the correct model.

Model selection through SINDy and IC ranking can be nuanced. As formulated in this text and [1], SINDy can be used on any problem for which the dynamics can be written as a sparse linear combination of numerically evaluated functions of the state variables. Trigonometric functions and other non-polynomial terms may be included. We also showed that PDEs can be inferred and evaluated using PDE-FIND [2]. In addition, we have shown that implicitly posed equations (i.e. $\Theta (\text{X},\dot{\text{X}})\xi =0$) can be inferred using a non-convex optimization in a framework we call implicit-SINDy [4]. This formulation was motivated by the rational functions that naturally result from separation of time scales and pseudo steady-state arguments in mass-action kinetic systems. In any of these frameworks, the number of terms in the model can still be calculated as the number of non-zero terms in the sparse coefficient vector. There are a large range of physical and biological systems where the possible mechanisms, and therefore functional forms are known, but combinatorial model selection is still difficult. SINDy and IC ranking provides a systematic and rigorous method to infer and evaluate suitable models in these cases.

When domain knowledge is insufficient to inform a suitable function library, generating appropriate models can be difficult. In some cases, SINDy can still find a representation of a system when the ‘true’ model is excluded from the library. For example, SINDy with a polynomial library can recover the Taylor-series expansion of a trigonometric function [1]. Alternative model generation/selection methods such as genetic algorithms can expand the form of library functions [6,38]. Regardless, the resulting models can still be compared using an information score because AIC does not require that the ‘true’ model be among those evaluated ([12], pp. 352–374). Therefore, AIC is the appropriate tool to compare models generated using different inference methods and to find a parsimonious model for the system without assuming any ‘true’ model exists. We believe this approach is well founded in the history of mechanistic modelling, whereby asymptotic analysis and other reduced-order modelling methods have sought to describe the dominant behaviour of a system rather than generate a complete or ‘true’ model.

In this work, we assume that all relevant state variables in the dynamics are measured. However, methods such as time-delay coordinates can be used to identify the dynamical structure of the system without measurements from all the ‘natural’ state variables [39]. Time-delay coordinates have been successfully combined with SINDy to recover low-order representations of the system [1,40]. Alternative approaches for identifying the presence of unmeasured, latent variables have been discussed in §2.2 of [5]. Further research into methods for identifying the relevant measurement variables to use during model selection is of broad interest.

Similar to other model selection or system identification methods, SINDy with AIC ranking will fail without sufficient data and when a low signal-to-noise ratio masks the sampling of the dynamics. If none of the library terms can sufficiently describe the data, or if inappropriate non-informative state variables are measured, SINDy will not generate a good (predictive and parsimonious) model. The future development of diagnostics for differentiating between these failure mechanisms is essential for application to real data. In conjunction with AIC, a suite of model validation tools will be required, including the evaluation of absolute error and other goodness-of-fit metrics. Domain-specific knowledge and modelling expertise are integral to these diagnostics, enabling implementation of SINDy and AIC on a variety of complex datasets.

The method presented provides an important contribution to standard model selection as well as to the SINDy paradigm. In particular, each of these methods has a significant shortcoming. In model selection, the shortcoming is centred around the inability of the standard AIC/BIC criteria to assess a combinatorially large set of candidate models. For SINDy, the sparse selection process for identifying the underlying dynamical systems lacks a principled method for selecting the correct dynamical model. The algorithm here circumvents both of these shortcomings. Specifically, the sparse regression of SINDy allows for the consideration of a combinatorially large number of candidate models. The sub-selected set of models can then each be evaluated using information criteria to select the correct dynamical system. The connection between information criteria and automatic model selection can also be integrated with genetic algorithms for selecting the structure and parameters of dynamical systems [6,24–26]. The process can be semi-automated for data-driven discovery of physical principles and laws of motion, which is now often referred to as the 4th paradigm of science [41].

## Data accessibility

This paper contains no experimental data. All computational results are reproducible and code is available at: https://github.com/niallmm/SINDy_AIC.
